# Real-world data from selective laser trabeculoplasty in Brazil

**DOI:** 10.1038/s41598-022-05699-6

**Published:** 2022-02-04

**Authors:** Ricardo Y. Abe, Heloísa A. Maestrini, Guilherme B. Guedes, Marcelo M. Nascimento, Camila I. Iguma, Hérika Danielle de Miranda Santos, Muna Georges Nasr, Ricarte P. Lucena-Junior, Tiago S. Prata

**Affiliations:** 1 Hospital Oftalmológico de Brasília, SGAS 607 Avenida L2 Sul, Distrito Federal, Brasília, ZIP Code 70200670 Brazil; 2 Department of Ophthalmology – University of Campinas, Campinas, Brazil; 3 Oculare, Hospital de Olhos, Belo Horizonte, Minas Gerais Brazil; 4 Hospital de Olhos do Paraná, Curitiba, Paraná Brazil; 5 Clínica Glaukos, São José do Rio Preto, São Paulo, Brazil; 6 Hospital Medicina Dos Olhos, Osasco, São Paulo, Brazil; 7 Departamento de Oftalmologia, UNIFESP/EPM, São Paulo, Brazil

**Keywords:** Medical research, Optic nerve diseases

## Abstract

Evaluate real-world data of outcomes from selective laser trabeculoplasty (SLT) performed in different regions of Brazil and investigate potential predictors of success associated with treatment. Multicenter retrospective case series with patients who underwent a primary SLT procedure. A total of 835 eyes from 835 patients were included. The mean follow-up was 916.8 ± 563.0 days. The mean age was 64.5 ± 14.9 years and 56.6% were women. We observed an intraocular pressure reduction comparing baseline to post-SLT measurements (18.4 ± 3.8 mmHg versus 14.8 ± 3.5 mmHg; P < 0.001) and mean number of glaucoma medications (1.8 ± 1.3 versus 1.4 ± 1.4; P < 0.001). We observed visual acuity loss over time (0.1 ± 0.3 versus 0.2 ± 0.3 logMAR, baseline and post-SLT, respectively, P = 0.009) and decrease in visual field mean deviation values (− 5.4 ± 5.9 versus − 5.7 ± 6.0 dB; P = 0.054) The Kaplan–Meier survival analysis showed an estimated probability of treatment success of 88% at 12 months, declining to 70% at 24 months and 54% at 36 months post-SLT. In the multivariable model, we found that a denser angle pigmentation (HR 0.69; 95% CI 0.57–0.85, P = 0.001) and corticosteroid treatment following SLT (HR 0.59; 95% CI 0.39–0.91, P = 0.018) were significantly associated with a lower risk for failure. Primary SLT achieved relatively high success rates without sight-threating complications in this real-world study with a large sample of Brazilian patients. These findings corroborate previous studies regarding SLT outcomes and may help clinicians to identify the best candidates for laser treatment.

## Introduction

Lowering intraocular pressure (IOP) is still the only available method to avoid the development of glaucoma or minimizing the risk of progression^1,2^. This IOP reduction can be achieved using different approaches such as eyedrops, laser and surgeries^3^. The concept of selectively targeting pigmented trabecular meshwork (TM) cells without damaging adjacent structures, using a q-switched 532-nm neodymium (Nd): YAG laser was described in vitro by Latina and Park in 1994^4^. Later, in a multicenter trial with 30 patients, Latina et al. showed that selective laser trabeculoplasty (SLT) was safe and effective in lowering IOP in patients with open angle glaucoma without coagulation of the TM^5^.

The use of laser treatment to lower IOP is not new, however the main advantage of SLT over conventional argon laser trabeculoplasty (ALT) is the absence of thermal damage to the TM, minimizing the risk of IOP spikes and peripheral anterior synechiae^5–7^. The low incidence of adverse effects of the procedure in conjunction with the IOP lowering capability has led to several clinical trials comparing SLT against eyedrops and also suggesting the use of SLT as a first-line therapy for open angle glaucoma and ocular hypertension^8–11^.

Recently a randomized controlled trial with 718 treatment-naive patients with open angle glaucoma or ocular hypertension was performed to compare initial treatment with eyedrops versus SLT^12^. Of 536 eyes treated with SLT, 509 eyes (95%) were at target IOP at 36 months. Whereas 499 (93%) of the 526 eyes treated with eye drops were at target IOP in the same period. The target IOP was achieved without need of medications in 419 (78%) of 536 eyes treated with SLT, most of them (76%) requiring only one laser treatment.

Data from real-world evidence generated during routine clinical practice obtained outside the context of controlled trials is relevant to corroborate findings from the latter studies. In fact, Khawaja et al. have recently performed a multicenter retrospective study with 831 eyes from 831 patients and found that SLT efficacy was better in patients with higher baseline IOP^13^. In other retrospective case series with 997 eyes from 677 patients, Kuley et al. found that greater baseline IOP and angle pigment was positively correlated with SLT success^14^.

In developing countries, glaucoma treatment can be challenging due to the scarcity of public resources, cost of medications and low accessibility to surgical options^15^. In addition, medication adherence to glaucoma treatment represents a barrier to avoid glaucoma progression^16,17^. In this scenario, SLT offers a cost-effective alternative to medical treatment^18,19^. Previous studies have investigated predictors of success and the use of SLT as first line therapy, but to date, there is no real-world evidence based on large SLT data obtained from Glaucoma Services in Brazil^20–22^. Such results would certainly add clinicians regarding best clinical indications and predictors of success for SLT in Brazilian patients.

The purpose of the current study is to evaluate real-world data of outcomes from SLT performed during clinical practice from different regions of Brazil and evaluate potential predictors of success associated with the procedure.

## Methods

### Participants

This was a multicenter retrospective study. We recruited participants from the Glaucoma Clinic at the Hospital Oftalmológico de Brasília, Glaukos Clinic in São José do Rio Preto, Oculare Clinic in Belo Horizonte, Hospital Medicina dos Olhos in Osasco and Hospital de Olhos do Paraná in Curitiba. The study protocol was revised and approved by the Institutional Review Board from the Hospital Oftalmológico de Brasília. All study methods complied with the Declaration of Helsinki guidelines for human subject research and informed consent from each patient was not required due to the retrospective nature of the study, and need for informed consent was waived by the Institutional Review Board from the Hospital Oftalmológico de Brasília. During follow-up, subjects underwent comprehensive ophthalmologic examinations including review of medical history, visual acuity, slit-lamp biomicroscopy, IOP measurement (Goldmann tonometer), gonioscopy (Posner goniolens), dilated fundoscopic examination with 78 diopters lens, and optic disc photography. Subjects underwent standard automated perimetry (SAP) using the 24-2 Swedish interactive threshold algorithm (Carl Zeiss Meditec, Inc, Dublin, CA) or Octopus 30-degree normal G2 visual field test (Octopus 600 perimeter, Haag-Streit AG, Koeniz-Berne, Switzerland). The SLT procedure was performed with appropriate laser gonioscopy lens, using standardized parameters such as 100 non-overlapping shots (25 per quadrant) and 360 degress of treatment with the exception in cases of pigmentary glaucoma in which 180 degrees and 50 non-overlapping shots were used. Laser energy varied from 0∙4 to 1∙3 mJ according to clinician discretion.

### Inclusion criteria

Patients who were submitted to the procedure were identified from the charts and screened for eligibility. For study inclusion, patients were required to be older than 18 years of age and had a minimum follow-up of 6 months post-SLT. In addition, patients were required to have had a baseline IOP assessment within 90 days before the index event. Only 1 eye was included per patient and for patients undergoing bilateral SLT we randomly selected one eye using simple randomization. The IOP data were collected in intervals such as: 7 and 30 days post-SLT and at 3, 6, 12, 18, 24, 30, 36, 42, 48, 54 and 60 months post-SLT.

### Outcome measures

The primary outcome of the study was to evaluate the SLT success rates based on change in IOP, number of glaucoma medications, need for SLT reapplications or other glaucoma surgeries for IOP control. As secondary outcomes we evaluated hazard ratios for treatment failure and complications from the procedure. In contrast to the study by Khawaja et al., we divided patients according to the indication of the SLT in 3 different common scenarios in clinical practice: uncontrolled IOP without medications, uncontrolled IOP with medications and controlled IOP with medications (patients who underwent SLT with the purpose of reducing the number of eyedrops). The definition for controlled or uncontrolled IOP was based on clinical assessment of the severity of the disease determined by the glaucoma specialist from each center.

Baseline IOP was defined as the mean value of the 3 last IOP measurement before the SLT procedure whenever these measurements were available to avoid regression to the mean effect. Among the 832 eyes, 571 (68.6%) had at least 3 IOP measurements and 717 eyes (86.1%) had at least 2 IOP measurements prior the procedure. We defined SLT treatment failure as 1 or more of the following: (1) the need for a subsequent glaucoma procedure, including repeat SLT; (2) IOP > 21 mmHg at the last visit or IOP reduction < 20% from baseline; or (3) an increase from baseline in the number of glaucoma medications at the last visit. Specifically, for the group with controlled IOP with medication, which the intention of the SLT treatment was reducing the number of eyedrops, we considered failure if the number of eyedrops was not reduced at the last visit. Cases in which another glaucoma procedure or SLT reapplication was required were also deemed as failures. Secondary outcomes of interest included changes in visual field mean deviation (MD) and visual acuity, and the use of additional glaucoma procedures post-SLT.

### Statistical analysis

Normality of the variables was assessed using the Skewness-Kurtosis test. Descriptive statistics included mean and standard deviation and Student’s T-tests for normally distributed (using one tailed test) and median, interquartile range and Wilcoxon rank-sum for non-parametrically distributed variables. We performed a Kaplan Meier survival analysis with log-rank test of equality across strata for the categorical variables and univariable Cox proportional hazard regression for continuous variables. The final multivariable model was built with variables with a p-value of 0.2 or less in the univariable analysis. To verify if the Cox proportional hazard model satisfies the assumption of proportionality, we checked proportionality by including time-dependent covariates in the model. Time dependent covariates are interactions of the predictors and time. In the current final model, all the time-dependent variables are not significant either collectively or individually thus supporting the assumption of proportional hazard. All statistical analyses were conducted with STATA, version 13 (StataCorp LP, College Station, Texas, USA). The alpha level (type I error) was set at 0.05.

## Results

We included a total of 835 eyes from 835 patients that underwent SLT procedure between the years of 2011 and 2020, with a mean follow-up of 916.8 ± 563.0 days. The mean age was 64.5 ± 14.9 years and 56.6% were women (Table 1). Within those that we were able to get data from ethnicity, 45.5% were White. Most patients had primary open-angle glaucoma (65.9%) and the most common indication for the SLT procedure was reducing eyedrops in eyes with controlled IOP (55%). Only 16.1% of the sample was naïve of glaucoma eyedrops.

**Table 1:** Clinical and demographic variables of subjects included in the study.

Parameters	Total subjects (n = 835)
Age, years	64.5 ± 14.9
Gender, % female	56.65%
**Race**	
% White	45.4%
% Black	7.8%
% Asian	3.3%
% Not informed	43.5%
Mean follow-up, in days	916.8 ± 563.0
Baseline IOP, mmHg	18.4 ± 3.8
Baseline IOP > 21 mmHg, yes	24.6%
Baseline mean deviation, dB	− 5.4 ± 5.9
Right eye , yes	56.0%
**Etiology**	
Primary open angle glaucoma	65.9%
Normal tension glaucoma	7.3%
Ocular hypertension	14.6%
Pigmentary glaucoma	4.6%
Primary angle closure glaucoma	2.8%
Pseudoexfoaliative glaucoma	1.3%
Pseudophakic, yes	42.8%
Previous ocular surgery, yes	3.1%
Pachymetry, μm	531.7 ± 31.7
**Degree of angle pigmentation**	
+/4	8.8%
++/4	45.4%
+++/4	33.3%
++++/4	10.5%
SLT performed at 360 degrees, yes	89.4%
**SLT clinical indication**	
Uncontrolled IOP without medication	16.1%
Uncontrolled IOP with medication	28.8%
Controlled IOP with medication	55.0%
Naïve of glaucoma eye-drops, yes	16.1%
**Type of glaucoma eyedrops in use**	
Prostaglandin analogues, yes	55.6%
Carboanhydrase inhibitor, yes	40.0%

*IOP* intraocular pressure, *dB* decibels, *μm* micrometers, *SLT* selective laser trabeculoplasty.

We observed a significant IOP reduction comparing baseline to post-SLT measurements (18.4 ± 3.8 mmHg versus 14.8 ± 3.5 mmHg; P < 0.001) and mean number of glaucoma medications (1.8 ± 1.3 versus 1.4 ± 1.4; P < 0.001) (Table 2). In addition, we observed best corrected visual acuity (BCVA) loss over time (0.1 ± 0.3 versus 0.2 ± 0.3 logMAR, baseline and post-SLT, respectively, P = 0.009) and decrease in MD values (− 5.4 ± 5.9 versus − 5.7 ± 6.0 dB; P = 0.054) (Table 2).

**Table 2:** Intraocular pressure, glaucoma medication, best corrected visual acuity and mean deviation before and after SLT.

Parameters	Baseline	Post-SLT	P value
Intraocular pressure (IOP), mmHg	18.4 ± 3.8	14.8 ± 3.5	< 0.001
Number of glaucoma eye-drops, mean	1.8 ± 1.3	1.4 ± 1.4	< 0.001
Best-corrected visual acuity, logMAR	0.1 ± 0.3	0.2 ± 0.3	0.009
Mean deviation, dB	− 5.4 ± 5.9	− 5.7 ± 6.0	0.054

*SLT* selective laser trabeculoplasty, *dB* decibels.

**Figure 1:**
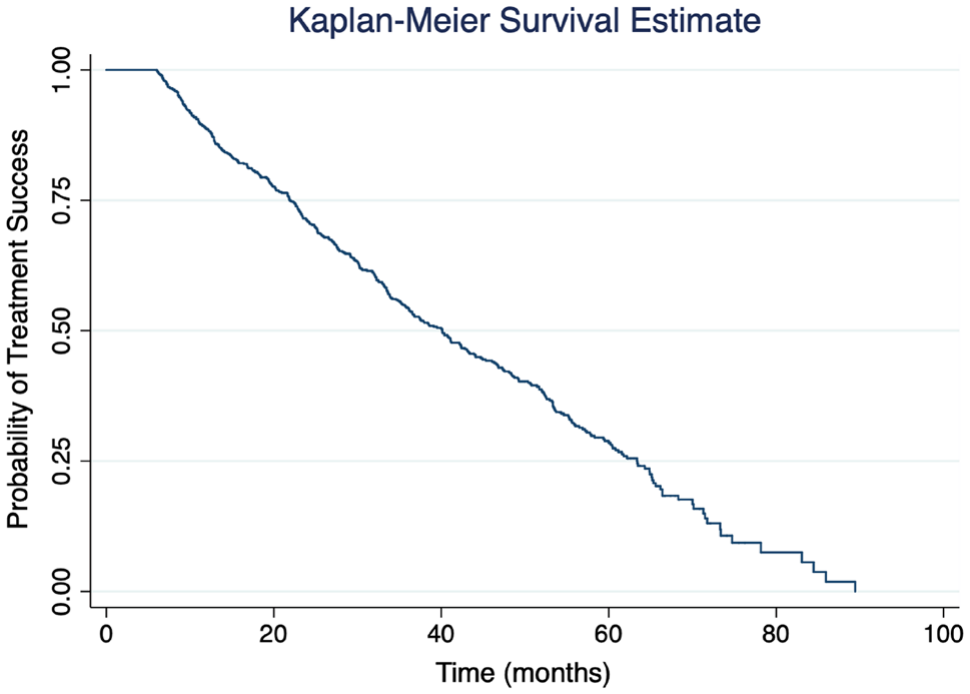
Kaplan-Meir survival estimates of eyes submittted to primary selective laser trabeculoplasty.

A total of 457 eyes (54.7%) presented failure during the follow-up, according to the criteria of failure described in the methods section. Among them, 73 eyes (15.9%) underwent SLT reapplication, and 70 eyes (15.3%) underwent another glaucoma procedure for IOP control during the follow-up. No sight-threatening complication was observed. From the total of 835 eyes that underwent SLT, only 20 eyes (3.7%), presented minor complications (13 eyes with IOP spikes > 5 mmHg and 7 eyes with persistent anterior chamber reaction). All complications resolved with clinical management without the need for further interventions. However, within these 20 eyes, 19 presented failures according to the criteria established in the study methodology. The Kaplan–Meier survival analysis showed an estimated probability of treatment success of 88% at 12 months, declining to 70% at 24 months and 54% at 36 months post-SLT (Fig. 1 and Table 3). We have also performed a separate analysis of survival according to groups. For patients with uncontrolled IOP without medications, the estimated probability of treatment success of 88% at 12 months, declining to 72% at 24 months and 60% at 36 months post-SLT. For patient with uncontrolled IOP with medications, the estimated probability of treatment success of 80% at 12 months, declining to 60% at 24 months and 45% at 36 months post-SLT. Finally, for patients with controlled IOP with medications (patients who underwent SLT with the purpose of reducing the number of eyedrops), the estimated probability of treatment success of 92% at 12 months, declining to 76% at 24 months and 55% at 36 months post-SLT.

**Table 3:** Estimated survival from the total sample of 835 patients submitted to primary selective laser trabeculoplasty treatment.

Time (in days)	Patients	Survivor function	Standard error	95% confidence interval	
06 months	835	0.9988	0.0012	0.9915	0.9998
09 months	754	0.9409	0.0083	0.9224	0.9552
12 months	672	0.8848	0.0113	0.8606	0.9051
18 motnhs	556	0.8026	0.0144	0.7725	0.8291
24 months	458	0.7106	0.0169	0.6760	0.7423
30 months	365	0.6318	0.0185	0.5944	0.6668
36 months	290	0.5437	0.0197	0.5042	0.5815
42 months	232	0.4768	0.0204	0.4363	0.5161
48 months	181	0.4193	0.028	0.3782	0.4597

We performed a univariable analysis to assess which variable is associated with the risk of SLT treatment failure (Table 4). Due to the retrospective design of the study, not all patients had complete information in the charts, therefore, we included the number of patients available in the column sample in Table 4, for each analysis performed. Patients with better baseline MD (less advanced disease) had better chances of obtaining success after SLT (hazard ratio [HR] 0.98 per dB; 95% CI 0.97–1.00, P = 0.006). Also, patients that underwent 360 degrees of SLT treatment presented lower risk of failures (HR 0.58; 95% CI 0.47–0.73, P < 0.001). Patients with dense angle pigmentation also presented lower risk of SLT failure (HR 0.66; 95% CI 0.54–0.80, P < 0.001). In addition, whereas patients that received corticosteroid eyedrops after SLT treatment presented lower risk for failures (HR 0.39; 95% CI 0.32–0.47, P < 0.001), patients that received nonsteroidal anti-inflammatory drugs (NSAID) after SLT presented higher risk for failures (HR: 2.54; 95% CI 2.06–3.13, P < 0.001).

**Table 4:** Univariable analysis of factors associated with failure after selective laser trabeculoplasty treatment.

Parameters	Sample	Hazard ratio	95% Confidence interval	P value
Age, per year	831	1.00	0.99–1.00	0.592
Ethnicity, black	472	1.35	0.79–2.31	0.261
Baseline IOP, per mmHg	835	0.98	0.95–1.00	0.140
Baseline MD, per dB	835	0.98	0.97–1.00	0.006
360 degrees treatment, yes	761	0.58	0.47–0.73	< 0.001
Eye, right	835	0.80	0.66–0.96	0.230
Pseudophakic, yes	835	0.88	0.73–1.06	0.280
Pachymetry, per um	835	0.99	0.99–1.00	0.828
Angle pigmentation ≥+++/4 , yes	795	0.66	0.54–0.80	< 0.001
Prostaglandin before-SLT, yes	828	1.04	0.86–1.26	0.634
Carbonic anhydrases before-SLT, yes	829	0.83	0.69–1.00	0.058
Naive of treatment, yes	832	1.02	0.81–1.30	0.808
Corticosteroid post-SLT, yes	768	0.39	0.32–0.47	< 0.001
NSAID post-SLT, yes	768	2.54	2.06–3.13	< 0.001

*IOP* intraocular pressure, *MD* mean deviation, *SLT* selective laser trabeculoplasty, *NSAID* nonsteroidal anti-inflammatory drug.

In the final multivariable model, a total of 767 patients were included (Table 5). We found that a denser angle pigmentation (HR 0.69; 95% CI 0.57–0.85, P = 0.001) and corticosteroid treatment following SLT (HR 0.59; 95% CI 0.39–0.91, P = 0.018) remained significantly associated with a lower risk for failure. This final model was evaluated to check proportional-hazards assumption proposed by Schoenfeld (P = 0.244)^23,24^.

**Table 5:** Multivariable analysis of factors associated with failure after selective laser trabeculoplasty treatment.

Parameters	Hazard ratio	95% Confidence interval	P value
Baseline IOP, per mmHg	0.99	0.96–1.02	0.646
Baseline MD, per dB	0.99	0.98–1.01	0.701
360 degrees treatment, yes	0.77	0.59–1.01	0.067
Angle pigmentation ≥ ++++/4, yes	0.69	0.57–0.85	0.001
Carbonic anhydrases before-SLT, yes	0.88	0.72–1.02	0.255
Corticosteroid post-SLT, yes	0.59	0.39–0.91	0.018
NSAID post-SLT, yes	1.48	0.96–2.28	0.071

*IOP* intraocular pressure, *MD* mean deviation, *SLT* selective laser trabeculoplasty, *NSAID* nonsteroidal anti-inflammatory drug.

## Discussion

This is the first real-world study in Latin America to report success outcomes from SLT and to evaluate possible risk factors for failure in a large sample of patients with glaucoma and ocular hypertension followed for a relatively long period of time. Several studies have showed previously that SLT can be offered as a first-line treatment for glaucoma and ocular hypertension patients, supporting a change of paradigm in clinical practice^9,12,25^. This is particular important, specially, in a developing country like Brazil, where access to public health services is scarce and the cost of treatment of the eyedrops may represent a barrier to adequate treatment adherence^17,26^. Thus, we believe that the findings of the present study, as derived from real-world data in a developing country, provides significant information regarding patients’ clinical course following SLT and may add clinicians to identify the best candidates for laser treatment, increasing the chances of successful management.

In the current study we divided the patients according to the SLT indication in clinical practice. Thus, 3 different scenarios were considered: First, patients with uncontrolled IOP without the use of medication, which is in line with that we currently offer to our patients as first line therapy. Second patients with uncontrolled IOP in which medications did not achieve the target pressure and SLT would be an option to reduce IOP or in some cases even delay a glaucoma surgery (if the patient was under maximum tolerated topical therapy). The third and last scenario is also common in clinical practice and consists of a group of patients that have IOP under control (either a glaucoma without progression with adequate target IOP with medication or a patient with ocular hypertension that achieved adequate IOP control with medication). This group of patients was submitted to SLT to reduce or eliminate eyedrops. In fact, in our sample most patients (55% or 456 eyes) were in this third group. Within this group of 456 eyes, 222 of them (49%) had a successful treatment at the end of follow-up. Additionally, 170 of them (37%) remained free of eyedrops during follow-up, showing that SLT is a good option to reduce the number of medications, improving adherence for the remaining bottles or even eliminating the need of eye drops, possibly leading to a better quality of life^27^.

There was a low prevalence of naïve to topical medication treatment patients in our sample (16%). This may have occurred due to our retrospective design, including patients since 2011 and at that time, offering SLT before introducing an eyedrop was not a consensus among glaucoma specialists. With the publication of several randomized clinical trials in recent years, such as the LIGHT study, scientific evidence has reinforced the concept and benefit of using SLT as a first line therapy and we expect that a higher number of patients will receive SLT before topical medication in Brazil^9,28^.

The current study showed an estimated probability of treatment success rate of 88% at 12 months, 70% at 24 months and 54% at 36 months after the SLT using the Kaplan–Meier survival analysis. This is the first study to show outcome results in a large sample in a Brazilian population. We should be careful when comparing these results with other real-world data studies, since the success rates will depend directly on the characteristics of the sample and also the criteria adopted to define failure or success. Khawaja et al. found significant reductions in IOP with treatment success in 70% and 45% of eyes at 6, and 12 months post-SLT, respectively. However, the majority failed treatment by 2 years (27% success at 24 months) due to an inadequate reduction in IOP (> 21 mmHg or < 20% reduction), or an increase in number of glaucoma medications, or by undergoing a subsequent glaucoma procedure. Kuley et al. in a study with 997 eyes from 677 patients found that that only 227 eyes (22.8%) achieved treatment success after 12-month follow-up^14^. These differences on success outcomes might have occurred since the majority of our patients (55% or 456 eyes) were submitted to initial SLT with an attempt to reduce or eliminate the use of eyedrops. It is important to highlight that even though for this specific group, we considered success the reduction of eyedrops, we still consider that the definition of success established were stringent since we still kept the other criteria for failure such as, the need for a glaucoma surgery, or new SLT to achieve target IOP or an increase in IOP values between baseline and last visit.

The investigation of predictors of success for SLT is important to guide the clinician into obtaining better outcomes and to provide patients with information regarding risks of failure and procedure outcomes. Despite previous studies have described baseline IOP as predictor of success, in our univariable and multivariable analyses, baseline IOP did not achieve statistical significance^14,29^. We found that patients with less advanced functional damage had better chances of obtaining success after SLT in the univariable analysis. This finding reinforces the concept that SLT is a good option for initial treatment, especially for those with mild glaucoma comparing to patients with moderate and advanced glaucoma that might require a lower target pressure. We also found that patients who underwent 360 degrees of SLT treatment presented lower failure risk comparing to 180 degrees of treatment. In fact, previous authors have already reported that performing 360 degrees is more effective than180 degreess^30,31^.

The influence of angle pigmentation in the outcomes of SLT is controversial. In the present study, the multivariable model showed that patients with denser angle pigmentation presented higher chances of treatment success, corroborating findings from previous studies^14,32^. However, Garg et al., investigating success predictors in the LIGHT trial, found that angle pigmentation was not directly correlated to absolute IOP reduction^28^. Latina et al. have described that coagulation of the TM is not an important component to the mechanism of IOP lowering after SLT^5^. In fact, disruption of pigmented TM cells appears to induce a response that results in a reduction of IOP probably by inducing trabecular cells hyperplasia with formation of healthy trabecular tissue and enhance outflow capacity^33^. Unfortunately, data from total energy used during SLT sessions was not available for most patients in our study. Therefore, a correlation between angle pigmentation and amount of energy was not performed.

To date there is no consensus on the optimal anti-inflammatory treatment regimen to be used after a SLT procedure. Comparing to ALT, SLT causes less inflammation since there is no thermal coagulation damage to adjacent cells of the TM^4^. Thus, in theory there is no need to use intensive anti-inflammatory drugs. In the present study, patients that received corticosteroid eyedrops after SLT treatment presented lower risk for failures (HR 0.59; 95% CI 0.39–0.91, P = 0.018) in the multivariable model. Unfortunately, we were not able to discriminate which specific type of steroid was used (prednisolone acetate or fluorometholone). Kim et al. evaluated the effect of anti-inflammatory treatment on the long-term (4.6 ± 3.4 years) outcome of ALT, comparing 0.25% fluorometholone versus placebo eyedrops four times daily before and after ALT^34^. They found no statistically significant differences in the success rate between groups. More recently, Jinapriya et al., performed a randomized, double-masked, placebo-controlled trial to compare prednisolone acetate 1%, ketorolac tromethamine 0.5% and placebo eye drops. They concluded that anti-inflammatory eyedrops after SLT does not seem to influence the IOP lowering effect of SLT compared to placebo^35^. However, a double-masked, randomized, placebo-controlled trial by Groth et al. showed that both NSAID and steroid treatment showed a statistically significantly greater IOP reduction compared with the placebo group after 12 weeks^36^. However, no difference was found between both anti-inflammatory agents. One could have hypothesized that in an uncontrolled setting such as this real-world study, patients with denser trabecular meshwork pigmentation undergoing SLT treatment would be more prone to receive an anti-inflammatory regimen with steroids eye-drops post-SLT, which could in part explain the fact that eye that received steroids had better outcomes as eyes with denser angle pigmentation also had higher success rates. However, as we performed a multivariable analysis, the use of steroids was independently associated with success treatment since the analysis was adjusted for angle pigmentation (Table 5). It is important to highlight that even though we found that eyes that received corticosteroid eyedrops regimen after SLT treatment presented lower risk for failures, more studies are necessary to evaluate the true effects of steroids and NSAID on SLT outcomes.

Even though the BCVA presented statistically significant decrease during follow-up and VF showed MD worsening with marginal statistically significance (Table 2), we believe that these changes might not be attributed specifically to SLT treatment. In fact, we must remember that glaucoma progression can occur even under regular treatment and patients might have developed media opacities, such as cataract during the follow-up. In addition, we do not believe that the amount of MD decrease could be considered clinically significant in the context of the length of the follow-up.

This study has several limitations. First, the findings from a retrospective study offers an inferior level of evidence comparing to randomized controlled trials. Second, data collection based on chart reviews often lead to missing information. For instance, in Table 4 we discriminate the number of patients included in each analysis and our final multivariate model included 767 patients (Table 5), which still represents most of our total sample. Nevertheless, this should be taken into consideration while interpreting our findings. On the other hand, it should be noted that the current study has a large sample size, from 5 centers in different regions of Brazil, being more representative of the general population and likely reducing the risk of selection bias. Third, the relatively low incidence of IOP spikes might be explained by the fact that we included the first IOP measurement only seven days after SLT treatment. Even though some centers included IOP measurements one day after the procedure, not all centers followed the same clinical routine. On the other hand, it should be noted that despite being derived from retrospective data, our rates of IOP spikes (1.5%) is very similar to the LIGHT trial findings (1.7%), as described by Garg et al.^28^.

This real-world study reported relatively high success rates without sight-threating complications following SLT in more than 800 Brazilian patients with glaucoma and ocular hypertension, followed for 30 months on average. We found that patients with denser angle pigmentation and those that received anti-inflammatory treatment with steroids after SLT had lower failure risk. Our real-world data not only corroborate previous findings regarding SLT outcomes, but also provides significant information regarding patients’ clinical course and may aid clinicians to identify the best candidates for laser treatment, reinforcing the change of paradigm in clinical practice in developing countries such as Brazil.
